# Light-Sensitive Vertical Migration of the Japanese Eel *Anguilla japonica* Revealed by Real-Time Tracking and Its Utilization for Geolocation

**DOI:** 10.1371/journal.pone.0121801

**Published:** 2015-04-15

**Authors:** Seinen Chow, Makoto Okazaki, Tomowo Watanabe, Kyohei Segawa, Toshihiro Yamamoto, Hiroaki Kurogi, Hideki Tanaka, Ken-ichiro Ai, Miho Kawai, Shin-ichi Yamamoto, Noritaka Mochioka, Ryotaro Manabe, Yoichi Miyake

**Affiliations:** 1 National Research Institute of Fisheries Science, Yokohama, Kanagawa, Japan; 2 Yokosuka Laboratory, National Research Institute of Aquaculture, Yokosuka, Kanagawa, Japan; 3 National Research Institute of Aquaculture, Nakatsuhamaura, Mie, Japan; 4 Chiba Prefectural Fisheries Research Center Freshwater Station, Sakura, Chiba, Japan; 5 Kagoshima Prefectural Fisheries Technology and Development Center, Ibusuki, Kagoshima, Japan; 6 Kyushu University, Fukuoka, Japan; 7 Nihon University, Fujisawa, Kanagawa, Japan; 8 The University of Tokyo, Kashiwa, Chiba, Japan; Institute of Marine Research, NORWAY

## Abstract

Short-time tracking (one to eight days) of the Japanese eel (*Anguilla japonica*) using ultrasonic transmitter was performed in the tropical-subtropical area adjacent to the spawning area and temperate area off the Japanese Archipelago. Of 16 eels (11 wild and five farmed) used, 10 wild eels displayed clear diel vertical migration (DVM) from the beginning, while the other five farmed eels tracked for 19 to 66 hours did not. During daytime, a significantly positive correlation between migration depth and light intensity recorded on the vessel was observed in the 10 wild eels, indicating that the eels were sensitive to sunlight even at the middle to lower mesopelagic zone (500 to 800 m). During nighttime, the eel migration depth was observed to be associated with the phase, rising and setting of the moon, indicating that the eels were sensitive to moonlight at the upper mesopelagic zone (<300 m). Two of 10 wild eels were in the yellow stage but shared similar DVM with the silver stage eels. Swimbladders of three silver stage eels were punctured before releasing, but very little effect on DVM was observed. The eels very punctually initiated descent upon nautical dawn and ascent upon sunset, enabling us to determine local times for sunrise and sunset, and hence this behavior may be used for geolocating eels. In fact, estimated positions of eels based on the depth trajectory data were comparable or even better than those obtained by light-based archival tag in other fish species.

## Introduction

The spawning area for the Japanese eel (*Anguilla japonica*) has been determined to be located at the southern part of the West Mariana Ridge due to having found matured and spent adult individuals and fertilized eggs [1–3]. The Japanese eel, therefore, must swim thousands of kilometers from the freshwater or near-shore growth habitats in northeast Asia to reach the above-mentioned open ocean spawning area without feeding [4]. However, information on migrating eels departing from the coastal area has been scarce. Using ultrasonic transmitters, diel vertical migration (DVM) (swimming at a shallower water layer in nighttime and at a deeper layer in daytime) of the European eel (*A*. *anguilla*) was observed in relatively deep areas of the Mediterranean Sea and Biscay Bay [5, 6]. The DVM of freshwater eels under oceanic migration, which has been considered to be a behavior primarily to avoid visually-oriented predators [7–12], was corroborated and much more clearly demonstrated by pop-up satellite archival transmitters (PSAT). On the other hand, such behavior was not observed for *A*. *anguilla* and *A*. *japonica* tracked in the spawning area using an ultrasonic transmitter, in which the maturing eels were suspected to prefer to stay in the shallow warm water [13, 14]. However, the tracking period was very short (<16 hrs), and the investigations were performed using a small number of farmed eels (*n* = 2 and 4), treated with hormone injection. Obviously, much longer tracking at different areas using wild eels is necessary for better understanding the eel’s swimming behavior.

The main objective of PSAT survey for freshwater eels is to investigate migration routes, since there has been no direct evidence on the migration routes of any freshwater eel species. PSAT may be a promising tool for investigating migration routes of fish, since the validity has been proven in large pelagic fishes such as tuna [15]. Nevertheless, all PSAT surveys for freshwater eels have been unsuccessful [7–12], since the eels dive toward the middle to lower mesopelagic zone during daytime where the PSAT could not collect measurable light intensity to estimate sunset and sunrise for geolocation.

During 2008 to 2010, Fisheries Agency and Fisheries Research Agency of Japan conducted research cruises in the southern part of the West Mariana Ridge in order to investigate spawning ecology of the Japanese eel, in which matured and spent adults of the Japanese eel (*A*. *japonica*) and giant mottled eel (*A*. *marmorata*) were first caught by RV Kaiyo-Maru, Fishery Agency of Japan [1, 2]. During these cruises, we performed tracking of eels with ultrasonic transmitters, aiming to investigate the swimming behavior of eels in the destination area (spawning area). In addition, we performed a similar tracking experiment around the Kuroshio Current off Japan in 2012, in order to collect information on the swimming behavior in the departure area (off nursery area). Tracking single fish by a large vessel is costly and restricted by ship-time, but fine grained data on the fish movement in relation to the dynamics of environmental changes may be obtained. We here introduce the results obtained in the research cruises and propose a promising tool for geolocating freshwater eels.

## Materials and Methods

### Ethics statement

The species *Anguilla japonica* was not an endangered species at the time of sampling. Wild eels were purchased from local fishermen possessing fishing licenses issued by Fishermen’s cooperative associations of the Tone River (Chiba Prefecture) and the Amikake River (Kagoshima Prefecture) and by Tokyo Metropolitan Government (the Naka River). Farmed eels were originated from glass eels caught under a commercial license issued by Aichi Prefecture.

### Eel specimens

**Fig 1:**
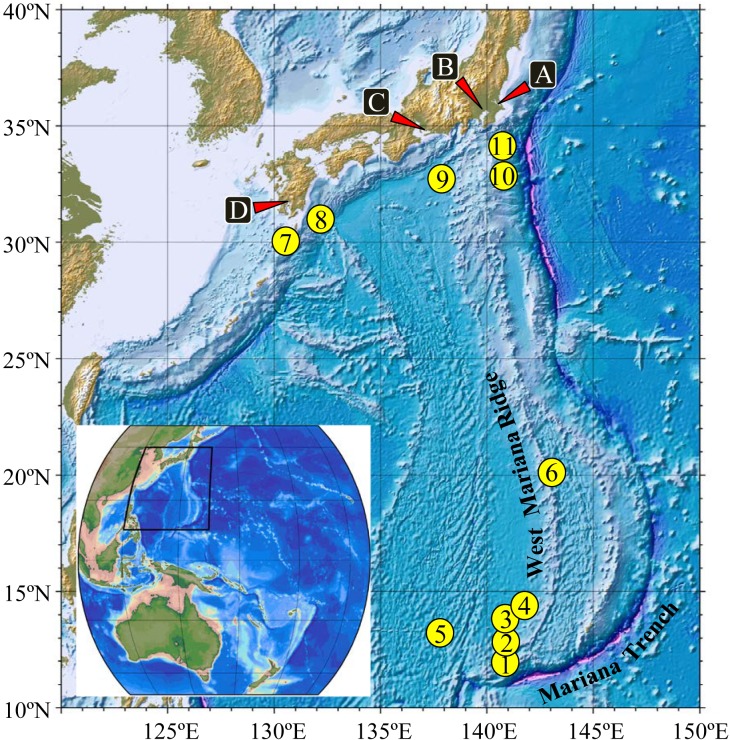
Four locations (A-D) for collecting the Japanese eels (*Anguilla japonica*) that were used for tracking, and eleven locations (1–11) where the eels were released and tracked. A: Tone River (Chiba Prefecture), B: Naka River (Tokyo), C: Mikawa Bay (Aichi Prefecture), D: Amikake River (Kagoshima Prefecture). 1–6: tropical to subtropical area (TS). 7–11: temperate area around Kuroshio Current (KC). See Tables 1 and 2 for details.

Information on the Japanese eels used for the tracking experiment is presented in Table 1, and the catch locations are shown in Fig. 1. Of 11 wild eels (WE), six were from the Tone River (A), four from the Naka River (B) and one from the Amikake River (D), Japan. Eels from the Tone River caught in September 2009 were kept in seawater at the Chiba Prefectural Fisheries Research Center at ambient temperature. Eels from the Naka River caught in October and November 2012 were kept in seawater at the National Research Institute of Aquaculture. An eel from the Amikake River caught in November 2012 was kept in freshwater at the Kagoshima Prefectural Fisheries Technology and Development Center for a week, and kept in seawater at the National Research Institute of Aquaculture for the next five days at ambient temperature. All five farmed eels (FE) were originated from glass eels caught in January 2004 at Mikawa Bay (C), and they were reared in freshwater for the first three years and in seawater for the next three years at the National Research Institute of Aquaculture at ambient temperature. All eels were transferred to RV Shoyo-Maru, Fishery Agency of Japan, on the day of or one day before departure, where wild and farmed eels were separately kept in two 200 L tanks at 20°C with circulation of filtered seawater and without feeding. Length, weight, and eye diameter of the eels were measured under anesthesia before attachment of transmitters in 2010, while those in 2012 were measured on land. Of 11 wild eels, eight were determined to be in the silver stage and three were determined to be in the yellow stage according to previously designated criteria [16].

**Table 1:** Information on 16 Japanese eels (*Anguilla japonica*) used in this study.

**ID**	**Origin**	**Location in Fig. 1**	**BL (cm)**	**BW (g)**	**Stage**
**WE2999**	Tone River, Chiba, September 2009	A	79.6	860	S1
**WE3001**	Tone River, Chiba, September 2009	A	77.5	740	S1
**WE3002**	Tone River, Chiba, September 2009	A	90.1	1,000	S1
**WE4263**	Tone River, Chiba, September 2009	A	70.5	660	Y2
**WE4264**	Tone River, Chiba, September 2009	A	77.5	580	Y2
**WE4265**	Tone River, Chiba, September 2009	A	72.2	460	Y2
**WE6286**	Naka River, Tokyo, November 2012	B	83.4	990	S2
**WE6287**	Naka River, Tokyo, November 2012	B	81.8	914	S2
**WE6288**	Naka River, Tokyo, October 2012	B	83.4	996	S2
**WE6289**	Naka River, Tokyo, October 2012	B	79.6	902	S2
**FE1**	Mikawa Bay, Aichi, January 2004	C	77.5	845	S1
**FE2**	Mikawa Bay, Aichi, January 2004	C	82.0	980	S1
**FE3**	Mikawa Bay, Aichi, January 2004	C	100.2	1,780	S1
**FE4**	Mikawa Bay, Aichi, January 2004	C	87.5	947	S2
**FE5**	Mikawa Bay, Aichi, January 2004	C	80.5	980	S1
**WE6285**	Amikake River, Kagoshima, November 2012	D	72.6	702	S1

**Table 2:** Summary of transmitter application and tracking for 16 Japanese eels (*Anguilla japonica*).

**ID**	**Location**	**Transmitter applied**			**Released at**				**Tracking ceased at**				**Period**
	**in Fig. 1**	**type**	**date**	**time**	**date**	**time**	**lat (N)**	**long (E)**	**date**	**time**	**lat (N)**	**long (E)**	**(h)**
**FE1** ^**a**^	1	P	June 10	10:30	July 9	22:53	12°00'	140°57'	July 10	21:00	12°00'	140°52'	22.1
**WE2999** ^**a**^	1	P	July 5	13:00	July 10	23:30	12°00'	140°59'	July 12	18:35	12°20'	140°54'	43.1
**FE2** ^**a**^	2	P	July 7	11:20	July 15	10:14	12°15'	141°00'	July 16	8:00	12°16'	140°47'	21.8
**WE3001**	3	P	July 9	12:45	July 13	0:25	13°00'	140°59'	July 14	19:10	13°08'	140°49'	42.5
**WE3002**	5	P	July 10	11:50	July 16	21:10	13°00'	138°00'	July 20	21:00	13°16'	137°18'	95.8
**FE4**	5	TP	July 16	9:30	July 21	2:38	13°00'	137°59'	July 23	21:00	13°02'	137°51'	66.4
**WE4263**	5	TP	July 23	21:40	July 24	1:10	13°00'	137°59'	July 26	20:00	13°11'	137°39'	67.8
**FE3**	5	P	July 11	9:40	July 26	0:20	13°01'	137°58'	July 27	19:28	13°02'	138°02'	19.1
**FE5**	5	P	July 11	10:10	July 27	22:55	13°01'	137°58'	July 28	22:55	13°04'	137°53'	22.5
**WE4264**	4	TP	Aug 3	14:30	Aug 8	0:35	14°15'	141°59'	Aug 14	20:00	14°29'	140°49'	163.4
**WE4265**	6	TP	Aug 15	17:10	Aug 15	20:18	20°00'	143°00'	Aug 19	22:10	19°37'	142°46'	97.9
**WE6285**	7	TP	Nov 26	17:00	Nov 28	21:27	30°02'	130°36'	Dec 3	21:30	30°29'	131°59'	118.0
**WE6288** ^**b**^	8	TP	Nov 26	17:20	Dec 4	21:13	30°58'	132°01'	Dec 6	21:13	31°45'	132°27'	49.0
**WE6289**	9	TP	Dec 4	12:30	Dec 7	22:42	32°49'	138°03'	Dec 15	22:42	32°30'	141°09'	191.0
**WE6287** ^**b**^	10	TP	Dec 9	13:00	Dec 16	5:19	33°03'	140°59'	Dec 17	20:00	33°54'	141°18'	38.2
**WE6286** ^**b**^	11	TP	Dec 16	12:00	Dec 17	23:17	33°57'	140°56'	Dec 19	23:17	34°55'	140°44'	48.0

^a^Since transmitters for FE1, FE2 and WE2999 were not calibrated, the depth data were not precise.

^b^Swimbladders of these wild eels (WE6286-6288) were surgically malfunctioned (SM eels).

### Ultrasonic transmitter application and study area

Seven depth-sensitive (P) and nine depth/temperature-sensitive (TP) ultrasonic transmitters (V16P and V16TP, VEMCO, Halifax, Canada) were used, and the data were transmitted at two and three second intervals, respectively (Table 2). The transmitter was 55 mm in length, 15 mm in diameter and weighed 9 g in water. Eels were kept in 10 L of seawater containing 0.1% 2-phenoxyethanol (v/v) for 15 to 30 min to anesthetize them. In fourteen eels (nine wild and five farmed), an approximately 4 cm length incision was made on the abdominal flank at middle position between gill slit and anus to surgically insert a transmitter in the body cavity. The incision was sutured with nylon string followed by quick-set adhesive. Swimbladders of three wild eels (WE6286-6288) (SM eels) were malfunctioned by cutting off a piece of the membrane 5 × 2 mm in size. Fifteen transmitters were activated upon surgery at several hours to 14 days before release, and one delayed-start transmitter (pre-programmed to activate 30 days after magnet removal) was inserted in one farmed individual (FE1). Since two wild eels (WE4263 and WE4265) appeared to be too small to insert a transmitter, the transmitter was attached in front of the dorsal fin using nylon string several hours before release. Visual investigation of the gonad upon surgery indicated that 14 were female, but the sexes of two small yellow eels (WE4263 and WE4265) were not determined.

Eleven eels (six wild and five farmed) were released and tracked at six locations (1–6) in tropical to subtropical area (TS), including the spawning area in summer 2010, and five wild eels were released at five locations (7–11) in the temperate area around the Kuroshio Current (KC) off Japan in early winter 2012 (Fig. 1, Table 2), while collecting environmental data. All eels were released at the surface; 14 eels were released during nighttime, one (WE6287) in the morning, and one (FE2) before noon. Since sea surface water temperature (ca. 29°C) was high in TS area, eels were kept in a separate container with 10 L of rearing water with aeration for several hours until the water temperature rose to ambient temperature. The container with an eel in it was hung alongside the ship and turned on its side to release the eel. Tracking was performed using SEA TRACK VP170-PC (VEMCO) equipped on RV Shoyo-Maru, where tracking at a maximum speed of 6 knots was possible. Areas where we performed eel tracking were deep, ranging from 1,809 to 5,254 m in TS area and 357 to 6,110 m in KC area. Tracking WE6285 in the shallow area in KC area (7 in Fig. 1) was very short, in which the sea-bed was 357 m at releasing position on the night of November 27 but the eel moved to a deeper area (>900 m) by the next morning.

### Environmental profiles

Temperature, salinity, and density profiles were collected by CTD (conductivity, temperature, and depth) probe (Sea-Bird SBE9) above the depth of 1,010 m at the positions of release and end of tracking, and occasionally those during tracking were collected by expendable CTD (XCTD). Light intensity was measured on the vessel, in which light intensity data at large range (~200 klux) during daytime was recorded using an illuminometer (N-149, Nippon Electric Instrument Inc., Japan), and that at small range (~20.0 lux) from 18:00 to 6:00 was recorded using a digital light data logger (TES-1336A, TES Electrical Electronic Corp, Japan).

### Data analyses

Simple correlation analysis was used to assess association between migration depth and light intensity during daytime. Migration depth data averaged every one minute were used, since light intensity data during daytime were recorded at one minute intervals. Correlation coefficient between migration depth and light intensity was evaluated by t-test. Geolocation of freshwater eels without light data may be possible using migration behavior as noticed and applied in the European eel [17, 18]. In order to determine the start points of large descent in the morning and large ascent in the evening, slope changes in the depth trajectory were first visually located. Subsequently, the depth data within 30 minutes before and after the time of this slope change were used for obtaining one minute averaged depth data. If the differences between these averaged depth data were consecutively positive for 10 minutes or longer, the behavior during this period was interpreted as an initial part of large descent, whereas it was interpreted as an initial part of large ascent if the differences were consecutively negative for 10 minutes or longer. The starting point of descent and ascent was defined as the time one minute before the start of this period. Based on the descent and ascent timings as determined above, sunrise and sunset times were estimated as described in Results and Discussion. Eels’ positions (latitude and longitude) were calculated by M-Series BASTrack software (Biotrack Ltd., Dorset, UK), which uses day/night length for latitude estimate and absolute time of local midday for longitude estimate. Sun altitude was set to -0.9. The estimated positions obtained were compared with actual eels’ positions at noon.

## Results and Discussion

### General view of vertical migration

**Fig 2:**
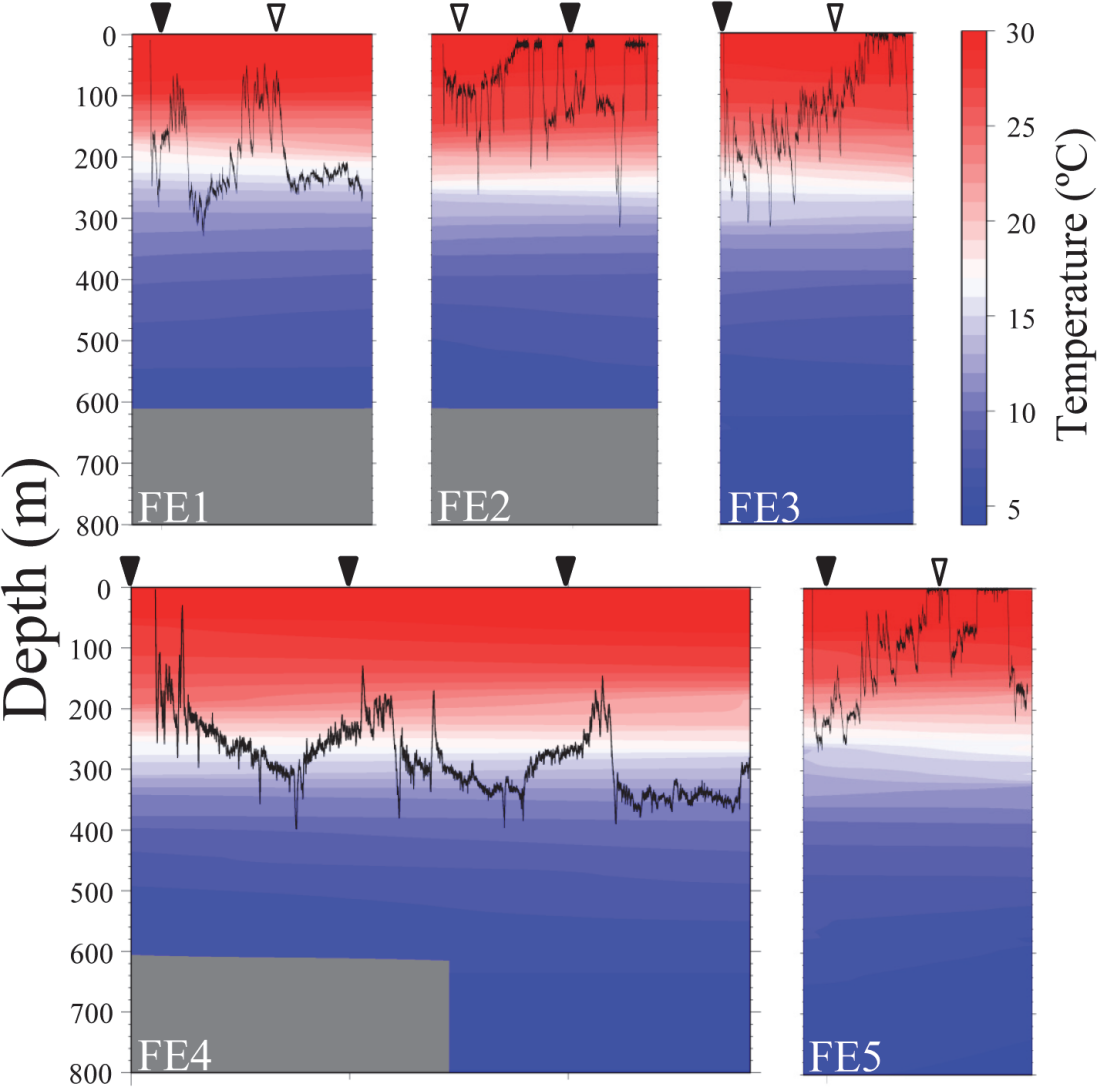
Vertical movement profiles of five farmed eels. The colors and contours indicate the water temperature. No temperature data was available for gray area. See Table 1 for individual information. Midnight (inverted closed triangle) and noon (inverted open triangle).

**Fig 3:**
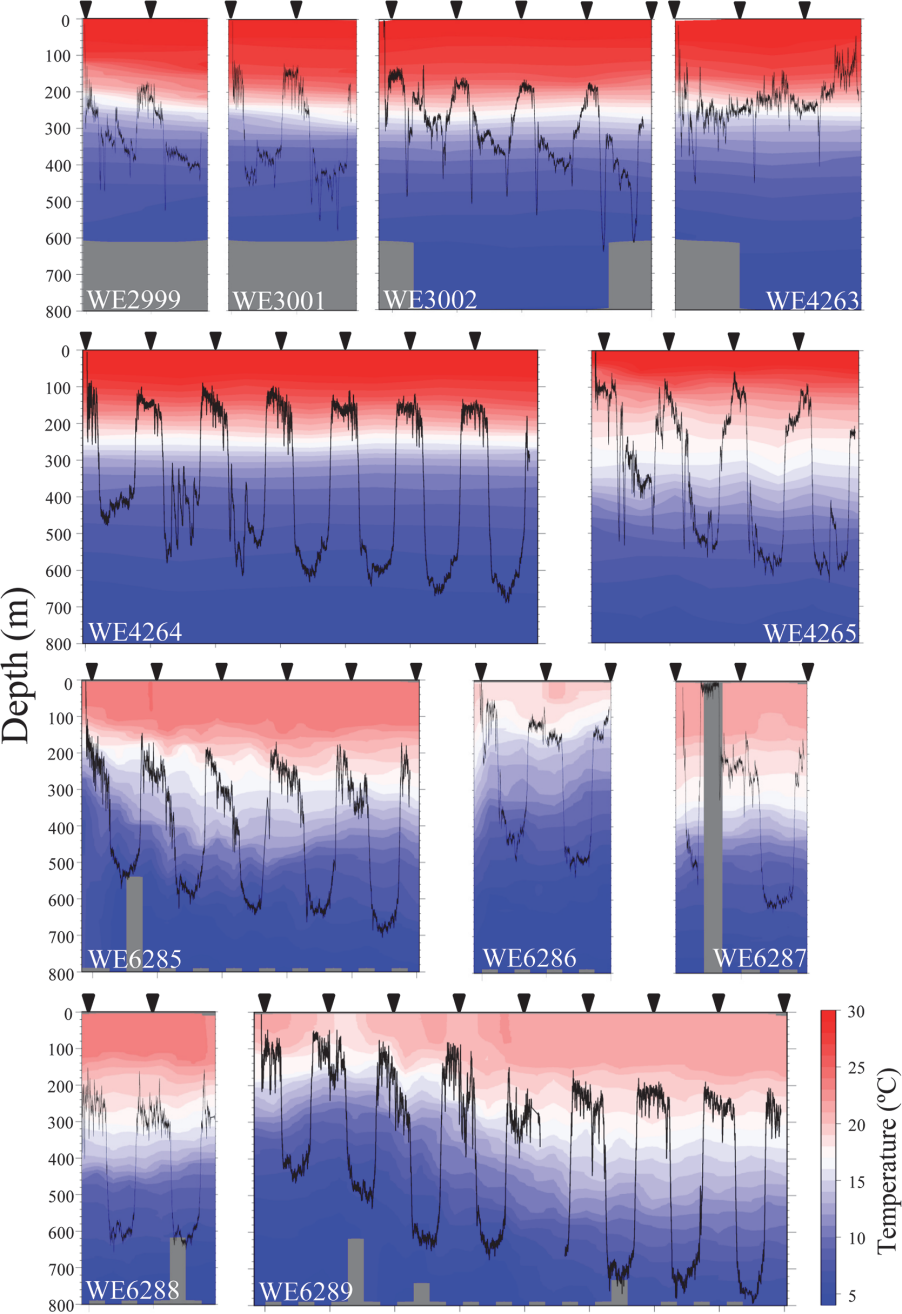
Vertical movement profiles of 11 wild eels. The colors and contours indicate the water temperature. No temperature data was available for gray area. See Table 1 for individual information. Midnight (inverted closed triangle).

All individuals quickly descended at a vertical speed of 29 to 73 cm s^-1^ upon releasing, reaching 100m to 300m deep within less than twenty minutes. Contrasting vertical migration behaviors between the farmed (Fig. 2) and wild (Fig. 3) eels were observed. All five farmed eels tracked for one to two days mostly stayed in the shallow layer (<300 m deep) even during daytime and did not show apparent DVM (Fig. 2). Since we witnessed the farmed eel FE5 swimming at the surface in the afternoon, the recorded swimming profile was not of a predator which had swallowed this individual. Clear DVM was observed in 10 of 11 wild eels, in which they descended upon dawn and ascended upon dusk (Fig. 3). We concluded that the behaviors of the farmed eels were not natural for at least the first two days after release. Therefore, the suppositions that maturing eels prefer shallow warm water and do not perform DVM [13, 14] might have resulted from using farmed eels. The smallest wild eel WE4263 (70.5 cm BL, Table 1) carrying a transmitter on its back descended upon dawn, but this individual did not show DVM within the subsequent 40 hrs (Fig. 3), suggesting that the weight and/or external attachment of a transmitter on the back may significantly affect the behavior specifically of smaller individuals. The effect of transmitter attachment, however, appears to vary as seen in another small wild eel WE4265 (72.2 cm BL, Table 1) carrying a transmitter on its back, albeit with somewhat unstable movement in the first two days. No further data analysis was performed for all five farmed eels and the smallest wild eel WE4263. Of the 10 wild eels exhibiting apparent DVM, one eel WE6287 (one of three SM individuals) showed peculiar movement in the first day (Fig. 3). This individual descended to 500 m deep in the first morning, started ascending before noon, surfaced and stayed in a very shallow layer (>50m) for five hours, but performed common DVM afterward. This peculiar movement observed at the very beginning might be attributable to the punctured swimbladder, but two other SM eels (WE6286 and 6288) had a common DVM profile (Fig. 3).

Speeds of descending at dawn and ascending at dusk were estimated to be 6.8 to 11.1 cm s^-1^ and 6.7 to 11.5 cm s^-1^, respectively, with no significant difference among individuals (Kruskal-Wallis test, P>0.5). The migration depth during daytime increased day by day, and the eels seldom ascended to a depth shallower than 400 m after two days of tracking (Fig. 3). Maximum daytime depth established in TS area was 690.2 m by WE4264 and that in KC area was 795.2 m by WE6289, both on the last day of tracking. The water temperature and dissolved oxygen at a depth of 600–800 m ranged from 5.0 to 7.8°C and 1.35 to 2.00 ml l^-1^ in TS area and 3.8 to 9.6°C and 1.19 to 2.11 ml l^-1^ in KC area. During nighttime, the eels usually stayed between the lower epipelagic and upper mesopelagic zones (150–250 m), where the water temperature and dissolved oxygen ranged from 14.4 to 26.7°C and 3.66 to 4.70 ml l^-1^ in TS area and 11.7 to 21.9°C and 2.21 to 3.30 ml l^-1^ in KC area.

### Daytime and nighttime migration profiles

**Fig 4:**
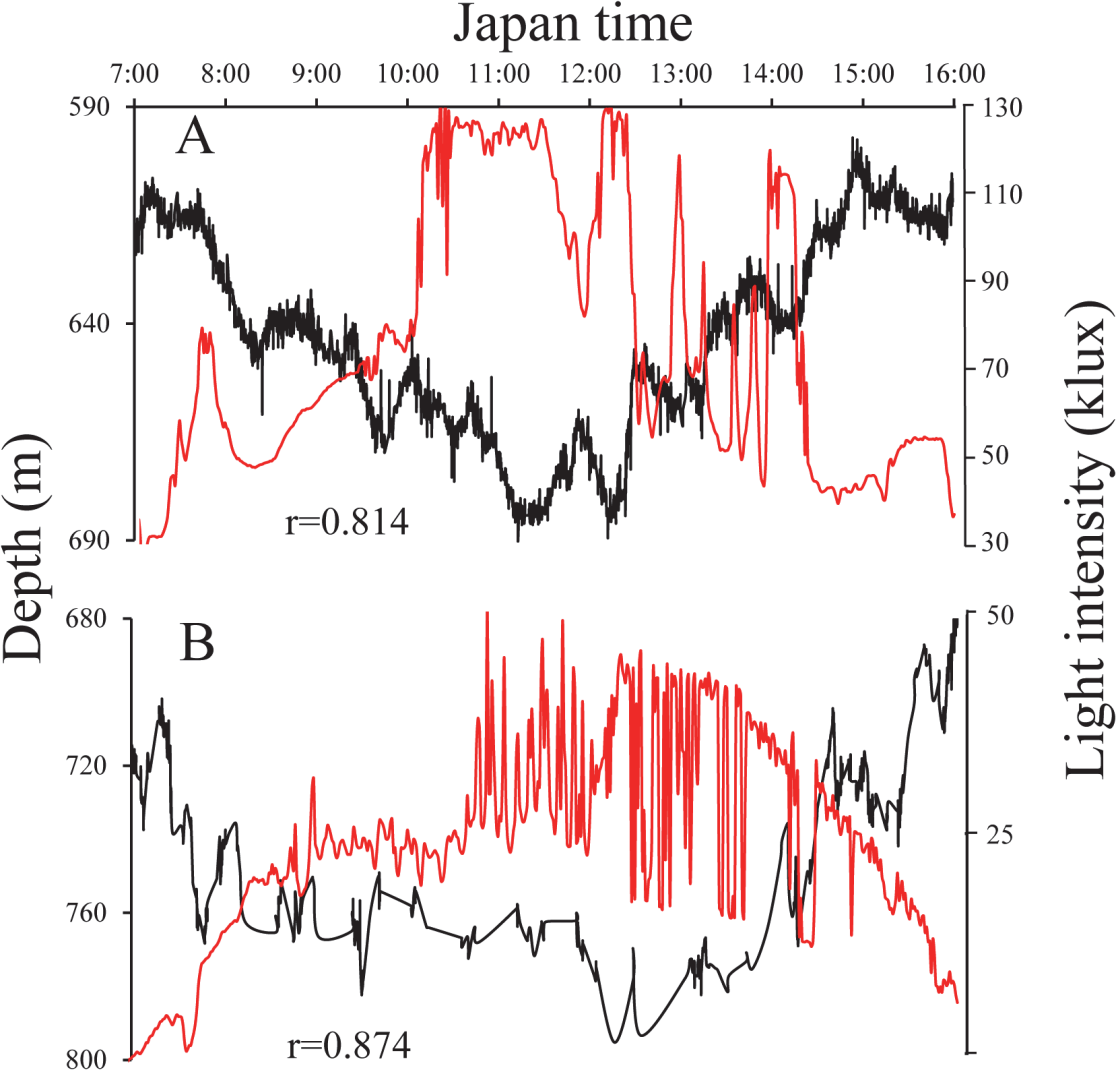
Vertical movements of eels during daytime (7:00–16:00) showing a reverse trajectory between migration depth (black lines) and light intensity (red lines) with a significantly positive correlation. A, WE4264 (August 14) (r = 0.814, P<0.01). B, WE6289 (December 11) (r = 0.874, P<0.01).

The two most striking migration profiles in relation to light intensity during daytime (7:00 to 16:00) are presented in Fig. 4. Clear reverse trajectories between migration depth and light intensity with significant positive correlations were found (r>0.8, P<0.01). Trajectories of migration depth of all eels and light intensity are presented in S1 and S2 Figs., in which a significant positive correlation between migration depth and light intensity (P<0.01) was detected in 10 of 19 observations in TS area (S1 Fig.) and 17 of 18 observations in KC area (S2 Fig.). Thus, eels’ responses to the change of light intensity were obvious even at the maximum depth (≈800 m) during daytime. Such reverse trajectories were not always evident, as negative (r<-0.23, P<0.01) and no correlations were observed in nine observations (A, C-F, H, I, P and S) in TS area (S1 Fig.) and one negative correlation (r = -0.456, P<0.01) (O) in KC area (S2 Fig.). Since our research vessel was tracking eels usually several hundred meters or more than one kilometer behind, actual light intensity above the eels may occasionally be different from that recorded on the vessel due to clouding. Although maximum light intensity and solar altitude at noon were much larger in TS area (≈130 klux and 90 degrees) than in KC area (≈50 klux and 40 degrees), upper and lower migration depths were generally greater in KC than in TS areas.

**Fig 5:**
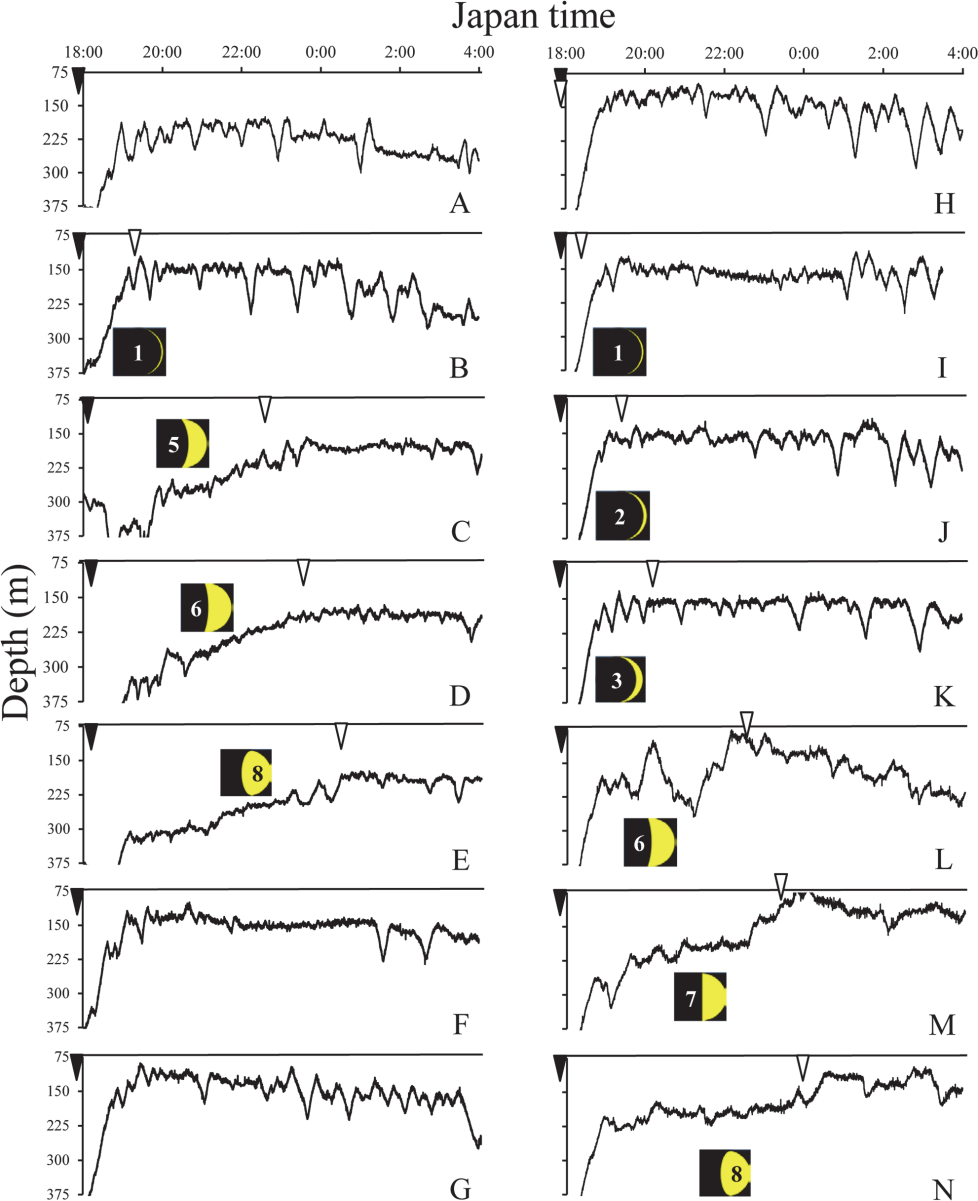
Vertical movement profiles (black lines) of five wild eels and lunar cycle during nighttime (18:00–4:00) in TS area. A, WE2999 (July 10–11). B, WE3001 (July 13–14). C-E, WE3002 (July 17–20). F-K, WE4264 (August 8–14). L-N, WE4265 (August 16–19). Moon phase is shown in the black quadrangle and is indicated by the number. Sunset (inverted closed triangle) and moon set (inverted open triangle).

**Fig 6:**
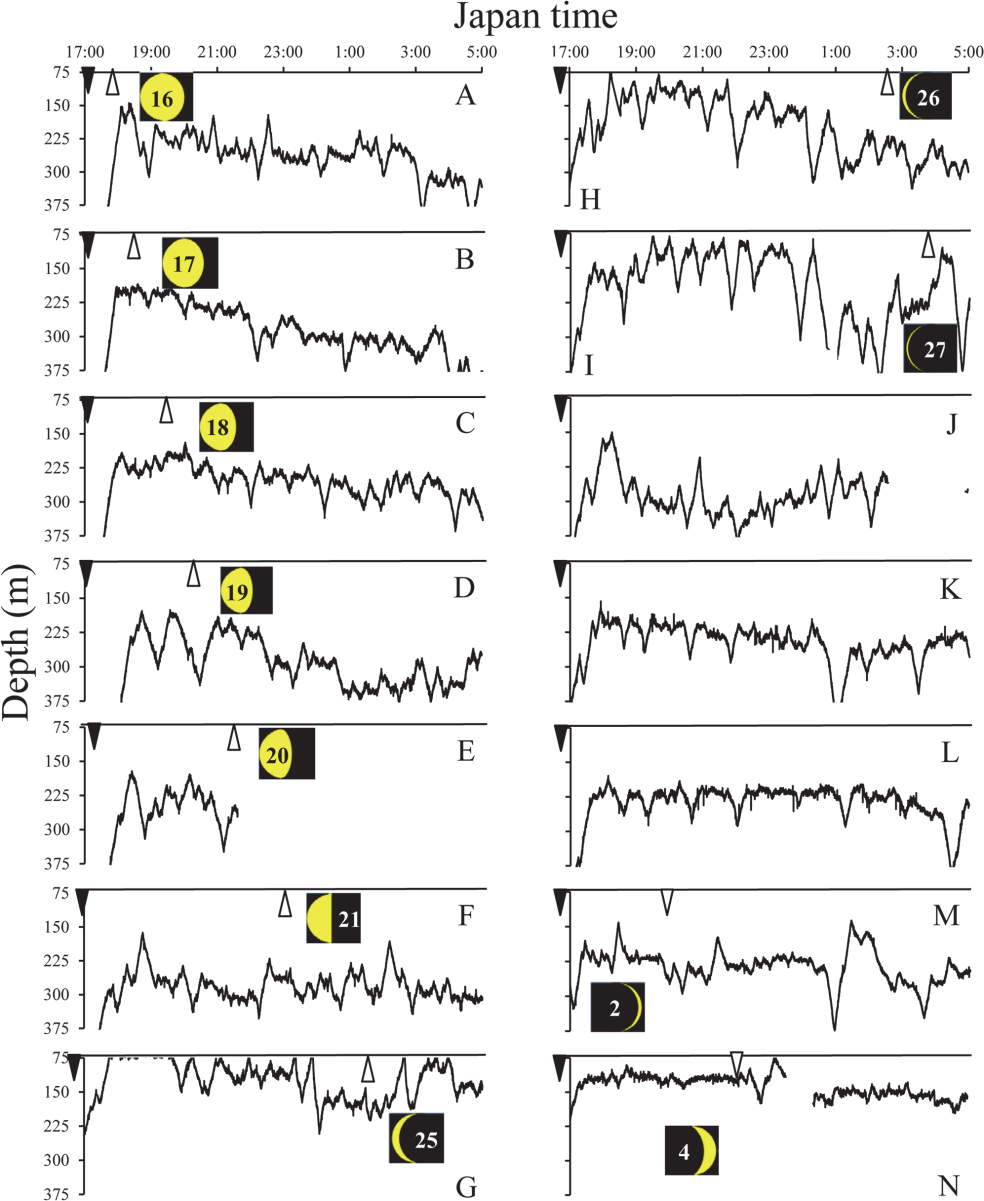
Vertical movement profiles (black lines) of five wild eels and lunar cycle during nighttime (17:00–5:00) in KC area. A-E, WE6285 (November 29—December 4). F, WE6288 (December 5–6). G-L, WE6289 (December 8–14). M, WE6287 (December 16–17). N, WE6286 (December 18–19). Moon phase is shown in the black quadrangle and is indicated by the number. Sunset (inverted closed triangle), moon rise (open triangle) and moon set (inverted open triangle).

Vertical movement profiles during nighttime (18:00 to 4:00) observed in TS and KC areas are presented in Figs. 5 and 6, respectively, showing their association with the lunar cycle. After sunset the eels smoothly ascended to the lower epipelagic to upper mesopelagic zones if there was no moon or a very small moon (Figs. 5A, B, F-K and 6), but they showed a descending tendency after moon rise when the moon was large (Fig. 6A-D). When a relatively large moon appeared after sunset, eels slowly ascended and reached the upper mesopelagic zone after moon set (Fig. 5C-E, L-N).

Since very similar DVM patterns in an oceanic environment have been observed in almost all freshwater eel species examined to date [7–12], DVM between upper and lower mesopelagic zones may be common and an inherent characteristic during the oceanic migration in freshwater eels. Avoidance from visually oriented predators is supposed to be a beneficial output from DVM for migrating eels [7–12], which may be corroborated by eels’ responses to light shown in the present study. The upper limit of nighttime migration depth in one *A*. *marmorata* was shown to be associated with the lunar cycle, in which median nighttime migration depth ranged from 230 to 269 m during a full moon while it ranged from 132 to 190 m during a new moon [12]. We could further reveal that nighttime migration depth and vertical movement of the Japanese eel were associated with the rising and setting of the moon and moon phase. To date, 15 adult eels (12 *A*. *japonica* and 3 *A*. *marmorata*) were captured in the spawning area using a large midwater trawl net [1–3]. Since very little information was available on the oceanic migration depth of the Japanese eel at the beginning of trawl survey [1, 2], the trawl net was towed based on the plankton net towing depth (0–500 m) for collecting eel leptocephali [19, 20] and water temperature (22–23°C) for eel larval rearing in the laboratory [21, 22]. However, the towing depth (163–303 m) of the large midwater trawl net [1–3] corresponded to the nighttime migration depths observed in previous PSAT surveys [7–12]. Since a successful catch was made in only nine out of 62 nighttime tows and all were around the new moon (four days before to two days after the new moon) [3], determination of net towing depth in consideration of the lunar cycle may lead to a more effective catch of migrating eels. Almost all freshwater eel species under oceanic migration have been observed to usually avoid the upper 100–150 m of the water column during the nighttime [7–12]. The water temperature seems to play much less a role for determining uppermost migration depth during nighttime. Because, depending on the area, water temperatures experienced by eels at the uppermost layer during nighttime were considerably different among species; ca. 12°C for *A*. *anguilla* [7], 17°C for *A*. *dieffenbachii* [8–10], ca. 26°C for *A*. *japonica* and *A*. *marmorata* [11, 12]. On the other hand, maximum daytime migration depth of all eel species corresponds to lower mesopelagic zone (800–1000 m), and the lowest temperature (ca. 4–5°C) recorded was similar among species [7–12]. The captive European eel (*A*. *anguilla*) was observed to enter torpor state at 1 to 3°C [23], and very few silver eels were observed to perform seaward migration below 4°C [24]. These suggest that the eels in the lower mesopelagic zone during daytime avoid deeper layers where they cannot maintain their physiological activity.

The European eel was observed to perform “steep” descent upon sunrise and ascent upon sunset [7], while the speeds observed in the present study were actually rather slow (6.7 to 11.5 cm s^-1^) but apparently sufficient for eels to migrate between the upper and middle mesopelagic zones (200–500 m) within an hour. Assuming that 0.5 to 1 kg eels may have 25–50 ml swimbladder [25], descending eels from the upper mesopelagic zone (200 m) have to fill 0.75–1.5 L of gas into the swimbladder to maintain neutral buoyancy at the middle mesopelagic zone (500 m). This value is thought to be unrealistic for daily vertical migration of the eels [26], since gas secretion rate in 50 cm silver phase American eels (*A*. *rostrata*) has been estimated to be 3 ml h^-1^ at the maximum [27]. Ascending eels may encounter a similar problem, as they have to excrete the same volume of gas from the swimbladder before reaching the upper mesopelagic zone. DVM profiles of SM individuals (having a punctured swimbladder) observed in the present study were comparable with those of the other individuals, and a peculiar movement was observed in one SM individual (Fig. 3, WE6287) only during the first daytime at shallow layer. Although striking functional, morphological and histological differences in the swimbladder were observed between yellow- and silver-stages of American and Japanese eels [27–29], two yellow-stage eels (Fig. 3, WE4264 and 4265) used in the present study shared similar DVM with silver-stage eels. These observations indicate that the migrating eels do not depend on the swimbladder to maintain neutral buoyancy specifically at depth as suggested previously [26]. Therefore, eels have to keep swimming in order to avoid sinking, and their depth control must primarily depend on hydrodynamic lift controlled by the angle of the enlarged pectoral fins. At shallower layer, on the other hand, the swelling swimbladder may act to gain buoyancy, and similar uppermost swimming depth (≈150–250m) shared by different eel species [7–12] might be partially determined by the pressure-swimbladder size relation. The effect of infection by swimbladder nematode *Anguillicola* on the oceanic migration of eels has been controversial [30, 31], but it seems to have little effect, at least on DVM. Nevertheless, the effect of malfunctional or less functional swimbladders for a much longer time span, such as oceanic migration, is totally unknown.

### Geolocation of eels using migration depth profile

**Fig 7:**
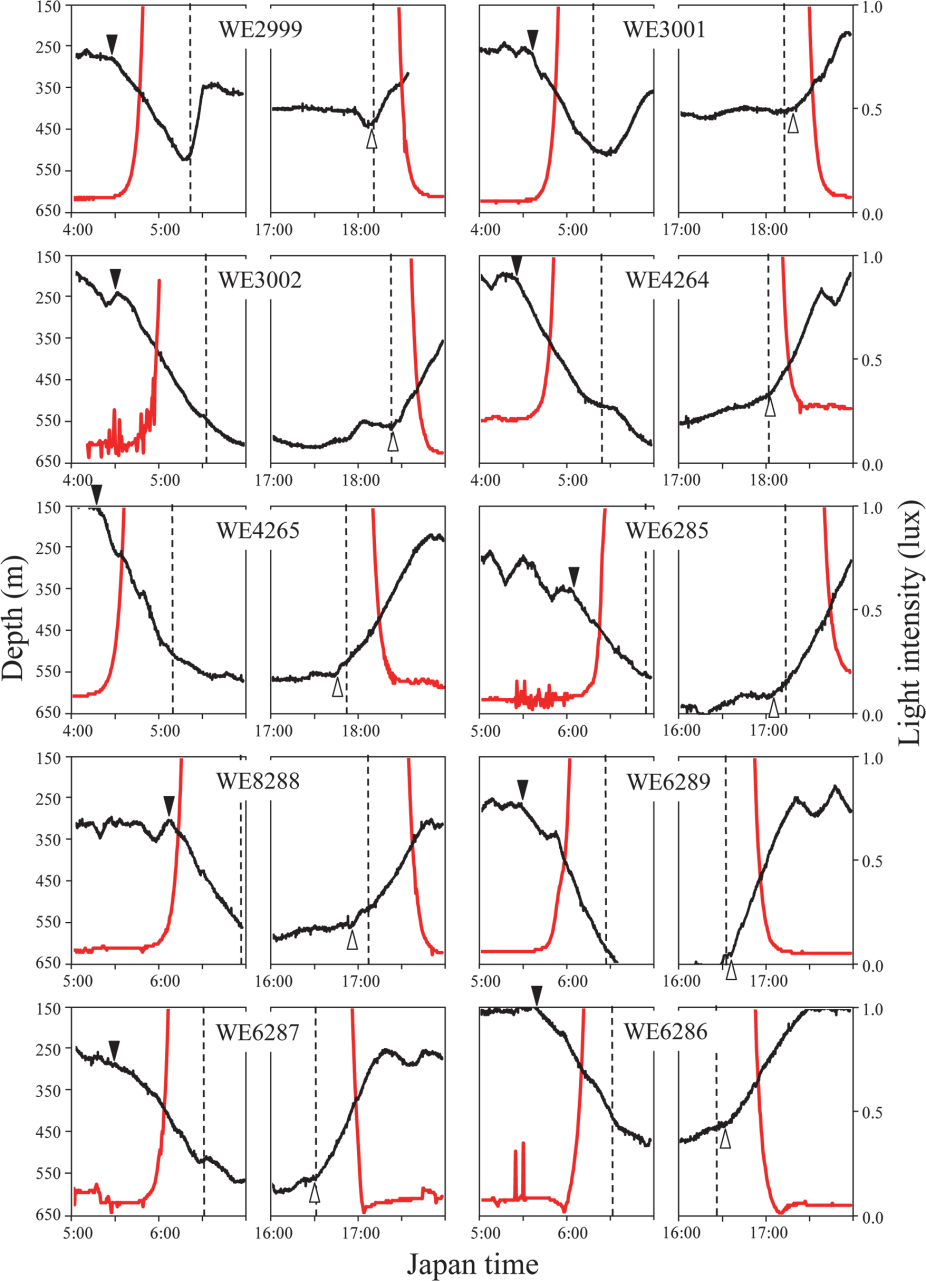
Vertical movement profiles of 10 wild eels in the morning (4:00–7:00) and evening (16:00–19:00) in the last day of tracking. WE2999 (July 12), WE3001 (July 14), WE3002 (July 20), WE4264 (August 14), WE4265 (August 19), WE6285 (December 3), WE6288 (December 6), WE6289 (December 15), WE6287 (December 17), and WE6286 (December 19). Black and red lines indicate eels’ migration depth and light intensity, respectively. Visually determined starting points of descent in the morning (inverted closed triangle) and ascent in the evening (open triangle). Vertical dotted line indicates sunrise or sunset.

**Fig 8:**
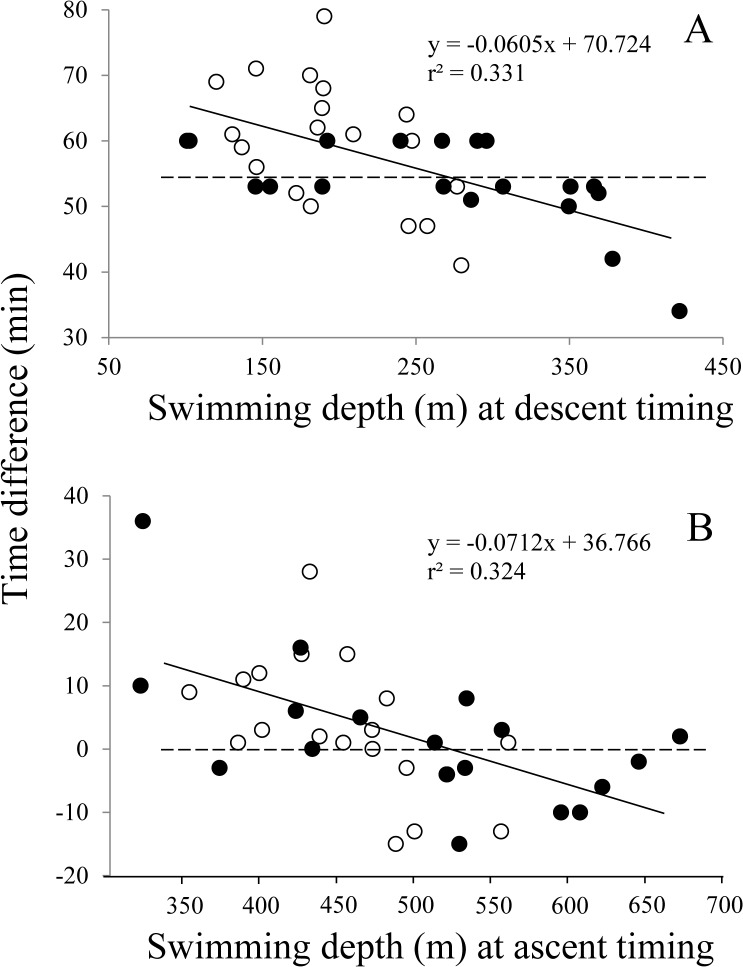
Time difference between descent timing and sunrise (A) and between ascent timing and sunset (B) plotted against migration depth at descent or ascent timing. Regression line is shown by a solid line. Dotted lines indicate 55 min difference in dawn and 0 min in dusk. Eels start descent approximately one hour before sunrise, corresponding to nautical dawn, in which individuals swimming at a shallower layer start descent earlier than those swimming at a deeper layer. Ascent in the evening is closely associated with sunset, in which individuals swimming at a shallower layer start ascent later than those swimming at a deeper layer. Observations in TS and KC areas are shown by open and closed circles, respectively.

Descending and ascending profiles in the morning (4:00 to 7:00) and evening (16:00 to 19:00) of all ten wild eels on the last day of tracking are presented in Fig. 7. These eels were observed to start descent approximately one hour before sunrise, approximately corresponding to the beginning of nautical dawn, while they started ascent upon sunset. Average difference between nautical dawn and descent timing was 3.2 (before nautical dawn) ±15.6 min, and those in TS and KC were 10.5 (before nautical dawn) ±9.8 min and 4.2 (after nautical dawn) ±10.9 min, respectively, with statistical difference between them (Mann-Whitney U test, P<0.001). Average difference between descent timing and sunrise was 56.7 (before sunrise) ±8.8 min, and those in TS and KC were 59.7±9.7 min and 53.7±6.8 min, respectively, with a slight but significant difference between them (Mann-Whitney U test, P = 0.04). Time difference between descent timing and sunrise was observed to decrease as eels’ depth at descent timing increased (r^2^ = 0.331, P<0.01), indicating that eels swimming at a shallower layer start descent earlier than those swimming at a deeper layer (Fig. 8A). Average difference between sunset and ascent timing was 2.3 (after sunset) ±10.9 min, and those in TS and KC were 2.9±10.8 min and 1.8±11.5 min, respectively, with no statistical difference between them (Mann-Whitney U test, P = 0.605). Significantly negative correlation was observed between the time difference and eel’s depth at ascent timing (r^2^ = 0.324, P<0.01), indicating that eels swimming at a shallower layer start ascent later than those swimming at a deeper layer (Fig. 8B). Thus, descent timing of eels at dawn relates to nautical dawn and ascent timing at dusk relates to sunset, but these timings are related to the migration depth.

**Fig 9:**
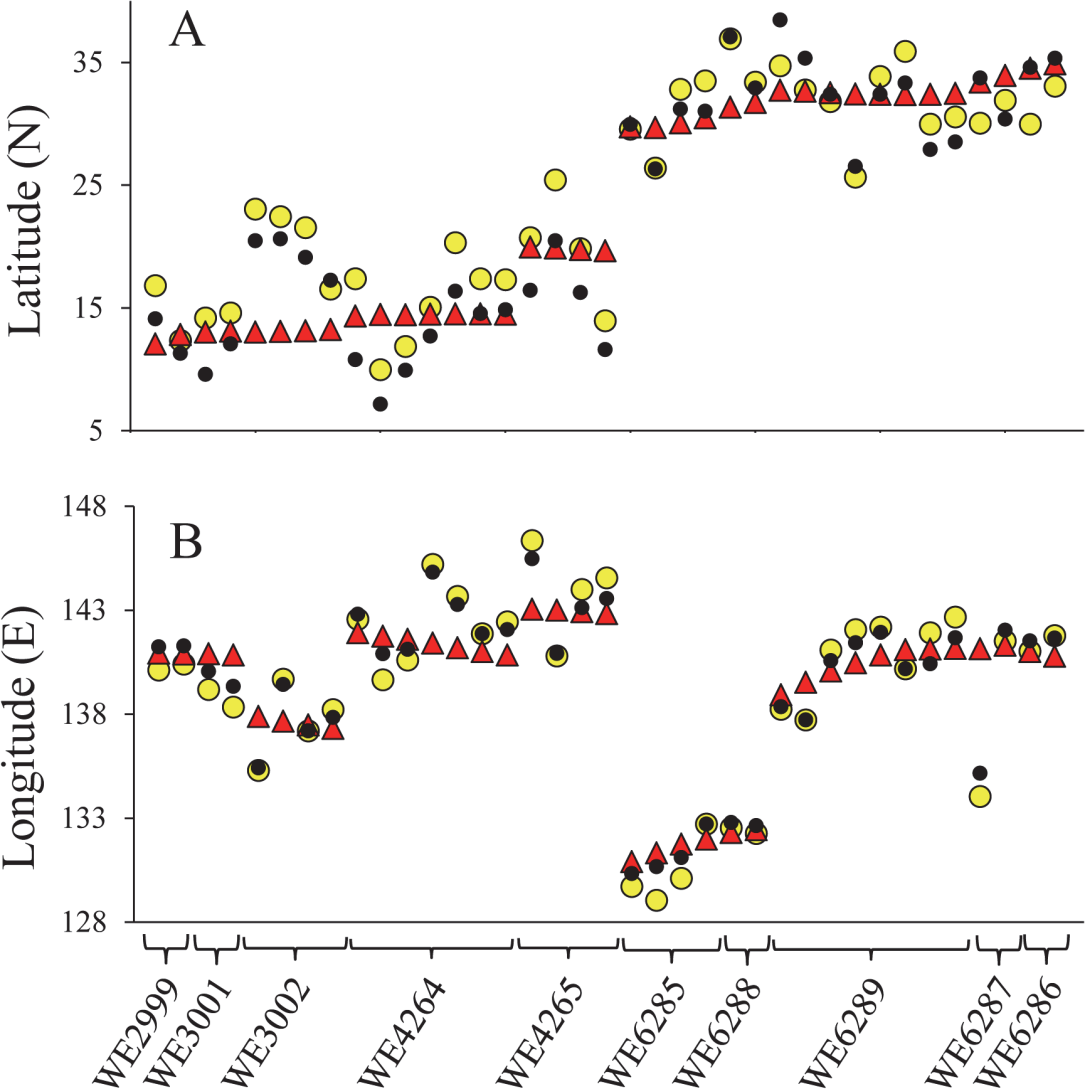
Geolocation of eels may be possible using depth data alone. Based on eel descent and ascent timings in the morning and evening, sunrise and sunset times were estimated and used for calculating latitude (A) and longitude (B) positions of ten wild eels. Actual eels’ position is shown by red triangle. Eel positions estimated by average and equation methods (see Results and Discussion) are shown by yellow and black circles, respectively.

Close proximity between swimming activity and nautical twilights was first observed in the European eel released at a very shallow area of the Baltic Sea [17]. Termination of swimming activity upon nautical dawn observed in the European eel [17] may be corresponding to descent timing at nautical dawn observed in the present study. On the other hand, onset of swimming activity was observed upon nautical dusk in the European eel [17], while ascent timing was corresponding to sunset in our study. This may be simply attributable to the eels’ swimming depth upon dusk. Spatio-temporal comparison of water temperature and depth data between the ARGO float and PSAT was performed in order to utilize PSAT data for geolocating eels [7], but the result was speculative and no matching profile could be obtained for determining longitudinal position. Given that light-responding activity-change profiles in the morning and evening observed in the present study, as well as in the previous study [17], are common for all freshwater eel species, geolocation for migrating eels may be possible only using depth data. Such an attempt was made with the European eel based on swimming activity profiles upon dawn and dusk, and longitude estimates obtained were reasonable [18]. However, the profiles of swimming activity in response to the light observed in shallow areas should not be directly applied to eels under oceanic migration, since the migrating depth is substantially different specifically during daytime. We performed two attempts to determine sunrise and sunset times based on observations in the present study (Fig. 8), in which sunrise was simply determined to be 55 min after the eels’ descent timing and sunset was assumed to be equal to the eels’ ascent timing (average method) and sunrise and sunset times were determined using the equation presented in Fig. 8 (equation method). Using these methods, the eels’ positions were calculated and compared with the eels’ actual positions at noon (Fig. 9). Average difference between the actual and calculated latitudes was -1.073±4.007° by average method and 0.294±3.853° by equation method (Fig. 9A). Average difference between the actual and calculated longitudes was 0.074±2.01° by average method and -0.027±1.585° by equation method (Fig. 9B). Variances in latitude were significantly larger than in longitude (F>3.6, P<0.005). In both latitude and longitude estimates, no statistical difference was observed between the estimates obtained by average and equation methods (Mann-Whitney U test, P>0.1), but estimates obtained by equation method were slightly closer to actual positions than those by average method. All estimates were comparable with, or even better than, those obtained in archival tag experiments in the other fish species [32, 33]. The larger error in latitude estimation may be inherent, as one min difference in estimating local noon results in 0.25° difference in longitude but the difference may be four times greater in latitude [32]. Water clarity and weather conditions may affect geographic positioning by light-based archival tags, and the same may be true for eels. Start timings of large descent and ascent correlate with the migration depth of eels, and the migration depth apparently correlates with not only the environmental factors mentioned above but also the lunar cycle and solar altitude. Nevertheless, the eels appeared to be much more sensitive to light than any existing light sensors equipped on archival tags, and our rough criteria presented in this study might be enough for illustrating a general view of a migration route in freshwater eels.

## Supporting Information

S1 Fig Vertical migration profiles (black lines) of five wild eels and light intensity (red lines) during daytime (7:00–16:00) in TS area. A, B: WE2999 (July 11 and 12), C, D: WE3001 (July 13 and 14), E-H: WE3002 (July 17–20), I-O: WE4264 (August 8–14), and P-S: WE4265 (August 16–19). Correlation coefficients (r) carrying an asterisk indicate a significant correlation between depth and light intensity (p<0.01). Among 19 observations, a significantly positive correlation was found in 10, whereas a significantly negative correlation was found in four (C, E, I and S) and no correlation in five (A, D, F, H and P).
(TIF)

S2 Fig Vertical migration profiles (black lines) of five wild eels and light intensity (red lines) during daytime (7:00–16:00) in KC area. A-E: WE6285 (November 29—December 3), F, G: WE6288 (December 5 and 6), H-N: WE6289 (December 8–15), O, P: WE6287 (December 16 and 17), and Q, R: WE6286 (December 18 and 19). Among 18 observations, a significantly positive correlation between depth and light intensity (p<0.01) was found in 17 and a significantly negative correlation was found in one (O).
(TIF)
