# Supplementary figures and images for: Light-Sensitive Vertical Migration of the Japanese Eel Anguilla japonica Revealed by Real-Time Tracking and Its Utilization for Geolocation
Source: PLoS One. 2015 Apr 15;10(4):e0121801. doi: 10.1371/journal.pone.0121801 (PMC4398447; doi:10.1371/journal.pone.0121801)

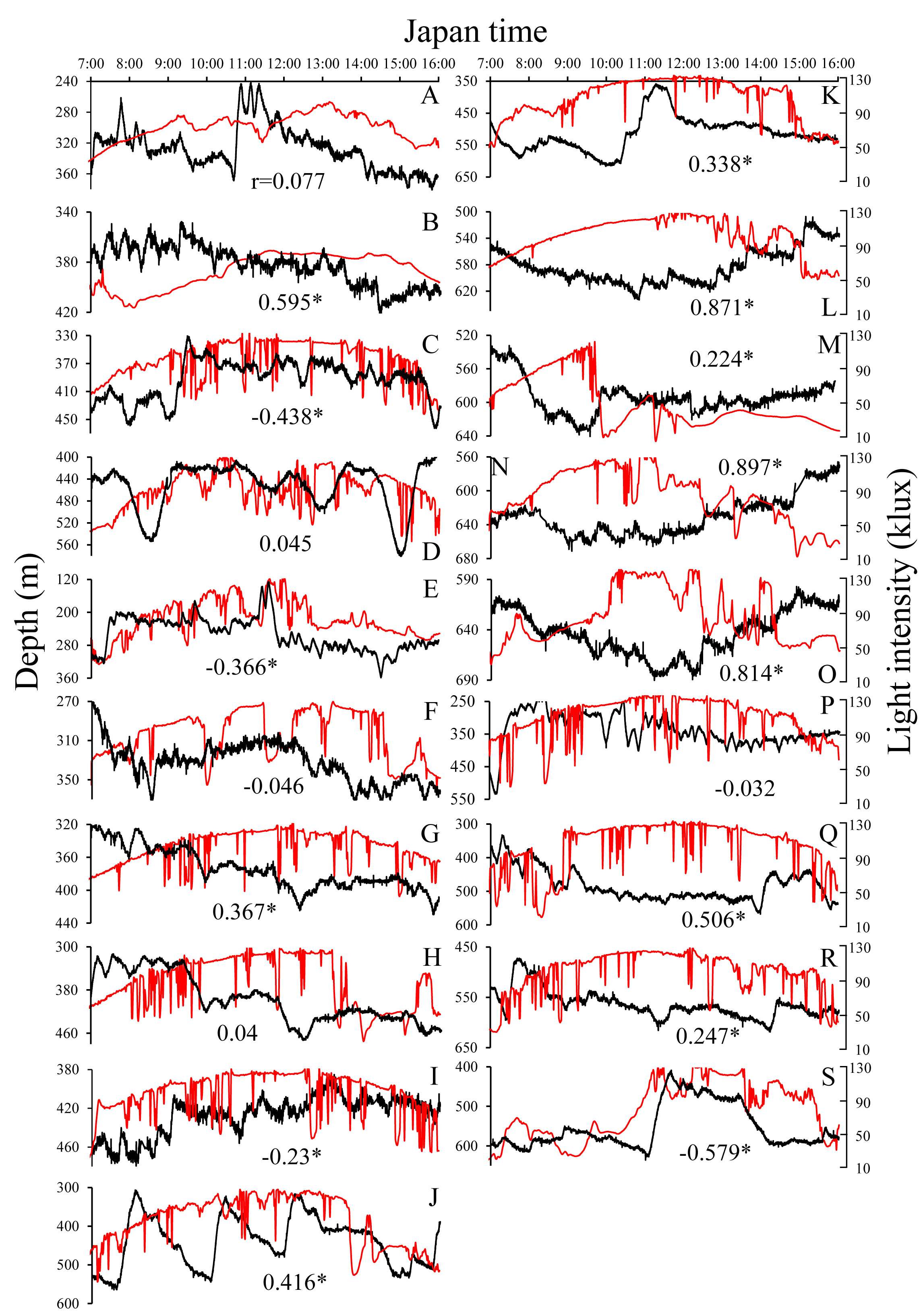

Supplement: S1 Fig — A, B: WE2999 (July 11 and 12), C, D: WE3001 (July 13 and 14), E-H: WE3002 (July 17–20), I-O: WE4264 (August 8–14), and P-S: WE4265 (August 16–19). Correlation coefficients (r) carrying an asterisk indicate a significant correlation between depth and light intensity (p<0.01). Among 19 observations, a significantly positive correlation was found in 10, whereas a significantly negative correlation was found in four (C, E, I and S) and no correlation in five (A, D, F, H and P). (TIF) [file pone.0121801.s001.tif]

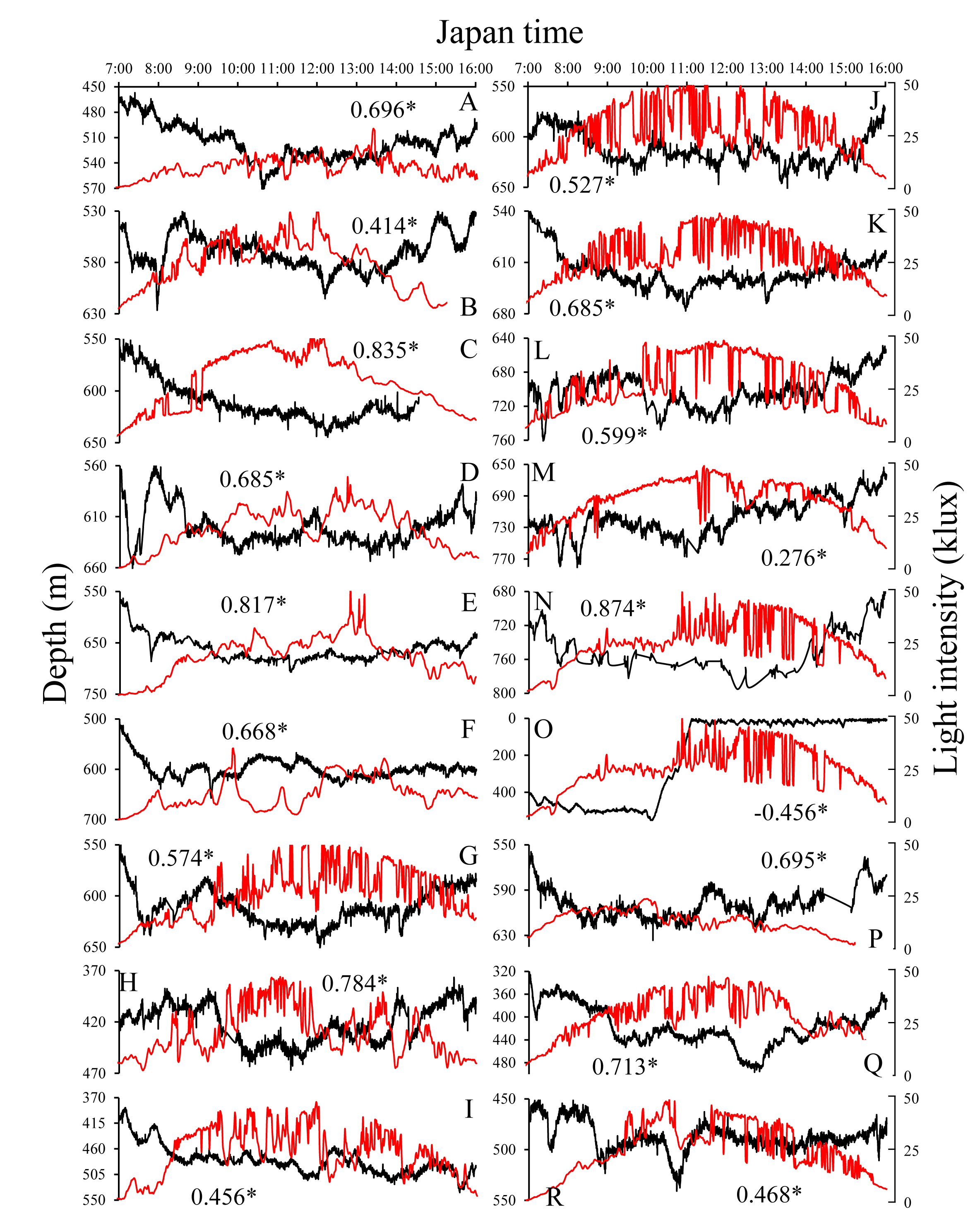

Supplement: S2 Fig — A-E: WE6285 (November 29—December 3), F, G: WE6288 (December 5 and 6), H-N: WE6289 (December 8–15), O, P: WE6287 (December 16 and 17), and Q, R: WE6286 (December 18 and 19). Among 18 observations, a significantly positive correlation between depth and light intensity (p<0.01) was found in 17 and a significantly negative correlation was found in one (O). (TIF) [file pone.0121801.s002.tif]
